# The Current Landscape of Pulmonary Hypertension in Myeloproliferative Neoplasms

**DOI:** 10.3390/cancers18172775

**Published:** 2026-08-26

**Authors:** Monica Ferrante, Hyeon-Ju Ali, Gabriela Hobbs, Orly Leiva

**Affiliations:** 1 Gill Heart and Vascular Institute, University of Kentucky College of Medicine, Lexington, KY 40536, USA; monica.ferrante@uky.edu; 2 Department of Cardiology, The University of Texas MD Anderson Cancer Center, Houston, TX 77030, USA; hrali@mdanderson.org; 3 Department of Hematology & Oncology, Mass General Brigham Cancer Institute, Massachusetts General Hospital & Harvard Medical School, Boston, MA 02114, USA; ghobbs@mgb.org

**Keywords:** myeloproliferative neoplasm, pulmonary hypertension, cardio-oncology, polycythemia vera, essential thrombocythemia, myelofibrosis, heart failure

## Abstract

Pulmonary hypertension (PH) is an increasingly recognized complication of Philadelphia chromosome-negative myeloproliferative neoplasms (MPNs). Recent data suggest prognostic implications of both cardiovascular morbidity and mortality and hematologic progression. Additionally, PH and MPNs share common pathophysiologic mechanisms that bridge these two seemingly different disorders. The landscape of PH in MPNs is evolving in the face of improved survivorship and reduced disease progression, leading to increased incidence and prevalence of concurrent and/or consequent cardiovascular disease. Prospective studies are necessary to refine screening protocols, risk stratification, and prognostic and therapeutic implications of PH in this population.

## Simple Summary

Pulmonary hypertension (PH), or elevated blood pressure in the pulmonary vasculature, is an increasingly recognized yet still underdiagnosed cardiovascular complication among patients with myeloproliferative neoplasms (MPNs). Currently, there are no standardized protocols for the diagnosis of PH in MPN patients. This review explores the mechanisms by which PH develops in MPN patients, how to approach screening, and the consequences on patient outcomes. Recent data suggest MPN patients with PH face up to a threefold higher risk of disease progression to leukemia and increased risk of major cardiovascular events (MACE). Whether earlier and systematic screening for PH in MPN patients is important is a question for future prospective studies.

## 1. Introduction

The Philadelphia chromosome-negative myeloproliferative neoplasms (MPNs) are a heterogenous group of clonal hematopoietic disorders that include polycythemia vera (PV), essential thrombocythemia (ET), and myelofibrosis (MF) [1]. Patients with MPNs are at increased risk of cardiovascular complications, including arterial and venous thrombosis, heart failure (HF), and pulmonary hypertension (PH) [2]. While the focus of cardiovascular disease research and clinician attention has been directed towards thrombosis, emerging evidence suggests that non-thrombotic cardiovascular complications (including HF and PH) are common and are associated with not only cardiovascular morbidity and mortality, but also hematologic progression and prognosis [2]. The landscape of cardio-oncology and MPNs is evolving in the face of earlier diagnosis, improved survivorship, and reduction in disease progression, ultimately driving increased incidence and prevalence of concurrent or consequent cardiovascular disease [3].

Pulmonary hypertension is an increasingly recognized complication across Philadelphia-negative MPN subtypes [2]. There is growing clinical salience given its prognostic implications for both major adverse cardiac events (MACE) and hematologic progression to secondary myelofibrosis (MF) or acute leukemia [3,4]. The clinical application of these novel associations is incompletely defined, in part due to the multifactorial and heterogenous phenotypes of both PH and MPNs. Further investigation is imperative to define optimal screening, diagnostic criteria, risk stratification, and optimal management.

### 1.1. Pulmonary Hypertension Epidemiology and Hemodynamic Classification

PH, as defined by the European Society of Cardiology/European Respiratory Society (ESC/ERS) 2022 PH Guidelines, requires a mean pulmonary artery pressure > 20 mmHg at rest on right heart catheterization (RHC) [5]. However, transthoracic echocardiography (TTE) remains the mainstay of noninvasive screening, risk stratification, and identification of patients warranting further evaluation with invasive hemodynamic evaluation [5,6]. PH is formally classified by the World Health Organization (WHO) groups according to etiology and hemodynamic parameters, and more broadly categorized as pre-capillary, post-capillary, or combined pre- and post-capillary depending on the primary driving mechanism [1,5]. Pre-capillary PH is caused by pathologic vessel remodeling or obstruction of pre-capillary pulmonary arterioles or small venules. Hemodynamically, it is defined as a mean pulmonary artery pressure (mPAP) > 20 mmHg, pulmonary capillary wedge pressure (PCWP) ≤ 15 mmHg, and a pulmonary vascular resistance (PVR) > 2.0 Wood units (WU) [5]. Isolated post-capillary PH, defined hemodynamically as PCWP > 15 mmHg and PVR ≤ 2 WU, is characterized by passive retrograde transmission of elevated left heart pressures due to systolic or diastolic dysfunction or valvular heart disease. Accordingly, management of pre-capillary PH may involve pulmonary vasodilators, whereas post-capillary PH management is directed at optimizing left-heart disease. Combined pre- and post-capillary PH (Cpc-PH) arises from either concomitant etiology of pre- and post-capillary PH or post-capillary PH with longstanding venous congestion leading to pathologic remodeling of pulmonary arterioles [5]. The hemodynamic profile of Cpc-PH is characterized by a mPAP > 20 mmHg, PCWP > 15 mmHg, and PVR > 2.0 WU, as seen in Table 1 [5].

**Figure 1:**
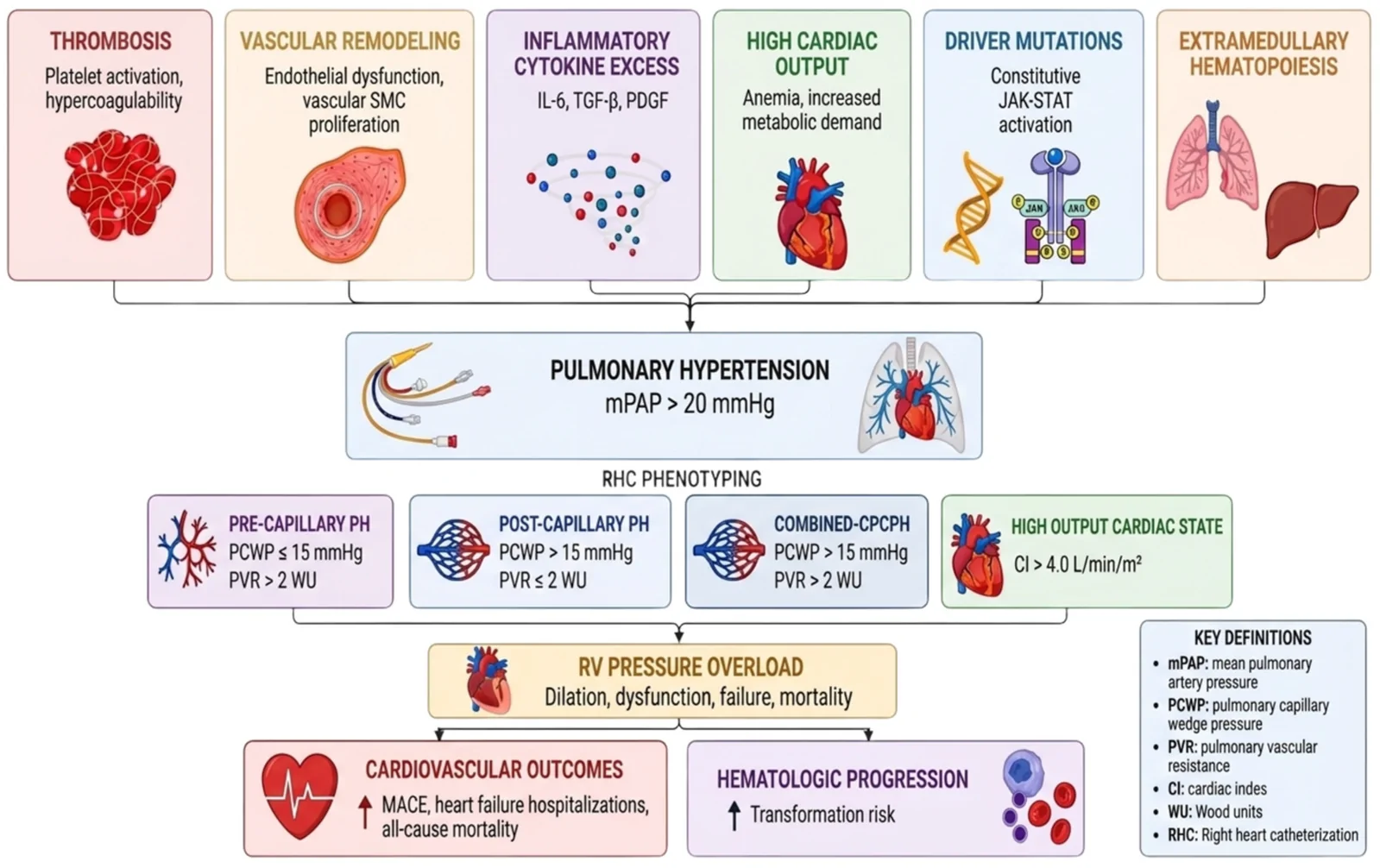
Overview of molecular mechanisms, hemodynamic phenotypes, and clinical outcomes of pulmonary hypertension in myeloproliferative neoplasms.

MPN-associated PH has classically been classified as group 5 (PH due to unclear and/or multifactorial mechanisms), though evolving data suggest there is significant heterogeneity within MPN-PH than historically recognized. In the French PH Registry, patients with MPN and severe pre-capillary PH were found to have either chronic thromboembolic disease (group 4 PH; 50%) or idiopathic PH, which was defined as group 5 (50%) PH. Among patients with ET and PV, chronic thromboembolic disease was more common, likely given their intrinsic prothrombotic nature [6]. Conversely, patients with MF were more likely to have group 5 PH, suggesting a different underlying pathophysiology. In addition to groups 4 and 5 PH, patients with MPN are also at risk of PH from LHD (group 2). Thus, patients with MPNs have a proclivity for both pre-capillary and post-capillary PH secondary to multiple risk factors, including thrombogenicity and heart failure [1,3]. In a study of 85 patients with MPN who underwent RHC due to concerns for PH, 70 (82.3%) patients had PH, of whom 30 (42.9%) had isolated pre-capillary PH, 12 (17.1%) had isolated post-capillary PH, and 28 (40.0%) had Cpc-PH [7]. This study highlights the importance of cardiovascular comorbidities such as coronary artery disease, hyperlipidemia, and hypertension, which can all contribute to heart failure secondary to diastolic dysfunction or ischemic heart disease (Figure 1).

Another potential etiology of PH among patients with MPNs is high-output heart failure (HOHF). HOHF is characterized by elevated filling pressures, signs and symptoms of HF, and elevated cardiac output [8]. Among patients with HOHF, MPNs account for 8% of cases, a disproportionately high representation when contextualized against the rarity of MPNs relative to other etiologies of HOHF such as obesity, liver cirrhosis, arteriovenous shunts, and lung disease, which are far more common [8]. Prior studies utilizing echocardiography to define HOHF, HOHF was present in 17% of patients with MPN [9,10]. In one registry, an elevated cardiac output greater than 7 L/min measured noninvasively with echocardiography correlated with a higher probability of PH and MPN disease progression [3]. Additionally, among patients with isolated post-capillary PH on RHC, HOHF is seen in over 40% of cases in MPNs [7]. However, the prevalence of HOHF and its clinical implications for outcomes among patients with MPN and PH remain fully characterized, though prior studies have demonstrated an association between HOHF and increased risk of MPN disease progression and adverse cardiovascular events [9,10].

### 1.2. Incidence and Prevalence of PH in MPNs

The prevalence of PH in MPNs varies widely in the literature due to the heterogeneity in diagnostic modalities (TTE vs. RHC) and pulmonary artery pressure cutoffs. In a meta-analysis of retrospective studies, the overall prevalence of PH in MPNs is estimated at 33%, with ranges approximated at 4% to 58% [11]. Recent studies that utilized TTE to define PH probability, either by defining echocardiographic probability of PH per guidelines or echocardiographic estimated pulmonary artery systolic pressure (PASP), have described an incidence of echocardiographic likelihood of PH between 24% and 43% [3,4,12,13]. Among patients with MPN, patients with MF have a higher incidence of echocardiographic probability of PH compared with ET or PV [4]. Similarly, the likelihood of echocardiographic probability of PH is associated with MPN disease duration [3,13].

## 2. Molecular Landscape and Pathophysiology

The pathophysiology of the association between MPNs and PH is not fully elucidated, though the mechanisms are likely multifactorial and mutually rooted in chronic inflammatory states. MPNs arise from acquired gain-of-function somatic mutations in the JAK-STAT signaling pathway, most notably *JAK2*, calreticulin (*CALR*), and myeloproliferative leukemia protein (*MPL*) [14,15,16]. Similarly, the development and progression of atherosclerosis, cardiovascular disease, and PH are associated with a dual pathogenesis of mutated *JAK2* pathway activation and non-mutational *JAK2* overactivation or cytokine-driven mechanisms [17,18,19,20].

The *JAK2V617F* mutation is the most common driver in MPNs and is found in 95% of patients with PV and 50% to 60% of patients with ET and MF [1,14]. However, *JAK2V617F* mutations can also be found in patients without any overt symptoms of MPNs and with normal hemograms in clonal hematopoiesis of indeterminate potential (CHIP) [21,22]. Constitutive JAK-STAT signaling creates a chronic inflammatory state partly mediated through proinflammatory cytokines, such as tumor necrosis factor α (TNF-α), interleukin (IL)-1B, and IL-6; profibrotic mediators, such as transforming growth factor β (TGF-β); and reactive oxygen species produced by mutant leukocytes and platelets in MPN. These cell signaling elements from *JAK2V617F*-mutated cells may elicit vascular remodeling characteristic of many PH groups [23]. Mutations in *JAK2* have also been associated with increased myocardial inflammation and accelerated heart failure in murine models [18].

Beyond inflammation, emerging evidence links *JAK2* mutations to maladaptive vascular remodeling, further supporting its role in cardiovascular and pulmonary vasculature pathology. Murine models with *JAK2V617F*-mutated neutrophils demonstrated increased infiltration in pulmonary vasculature and accelerated remodeling due to JAK-STAT up-regulation. Similarly, preclinical studies linked PH and MPNs by demonstrating an association between elevated levels of bone marrow-derived CD34^+^/CD133^+^ endothelial progenitor cells, adverse pulmonary vasculature remodeling, pre-capillary PH, and right heart failure [4,23]. In mouse models of PH due to chronic hypoxia, *JAK2V617F*-mutated clonal hematopoiesis led to accelerated PH and increased pulmonary vascular remodeling [19]. Interestingly, this adverse pulmonary vascular remodeling was mediated in part by increased perivascular *JAK2V617F*-mutated neutrophil infiltration. There was an upregulation of activin A receptor type 1 (ACVRL1) among mice with the *JAK2V617F* mutation that also mediated the development of PH in this murine model [19]. The upregulation of ACVRL1 is particularly of clinical importance since ACVRL1 is implicated in the progression of PH, and a novel targeted PH-specific therapy, sotatercept, exerts its effect on PH via inhibition of this pathway [24,25,26].

The inflammatory, profibrotic, and prothrombotic nature of *JAK2* mutant hematopoietic cell clones may predispose patients to CTEPH (group 4) or group 5 (MPN-associated PH). Extramedullary hematopoiesis (EMH) in the pulmonary vasculature, most seen with MF, may cause direct vessel obstruction and negative remodeling or precipitate anemia, increased catabolic activity, HOHF, and consequent PH. Predisposition to venous thromboembolism in MPNs may impart greater risk for CTEPH, as can splenectomy due to its increased VTE risk. MPN patients also carry a predisposition to post-capillary PH (group 2) owing to increased risk for LHD, such as heart failure and coronary artery disease. Conversely, the mechanisms by which PH or HF may contribute to accelerated hematologic progression are less established, though may be related to the mutual *JAK2*-driven inflammatory milieu with positive feedback from cardiovascular stress response. However, prospective studies are needed to establish the relationship between PH and HF and hematologic progression.

Mutations in other driver genes, including calreticulin (*CALR*) and myeloproliferative leukemia virus (*MPL*), are present in approximately 30% to 40% of patients with ET or MF [15,27]. Less is known about the impact of *CALR* and *MPL* on the development of PH and HF in MPNs, though all driver mutations in MPN lead to constitutive activation of the JAK-STAT pathway. In mouse models, mice with *CALR* knock-in mutations demonstrated more pulmonary vascular remodeling and elevated right ventricular systolic pressures compared with control mice [28]. This study also demonstrated increased STAT3 activation under hypoxic conditions in the *CALR*-mutated mice compared with wild-type. In a study of 247 patients with MPNs without elevated PASP (<40 mmHg) on initial TTE who had repeat TTE at least 12 months apart, non-*JAK2* driver mutations (*CALR*, *MPL*, or triple-negative status) were associated with increased risk of elevated PASP on subsequent TTE compared with those with *JAK2* mutations [29]. Patients with MF and triple-negative mutational status and ET with *MPL* mutations have been associated with increased risk of hematologic progression, which may partially explain the association between these mutations and the risk of incident elevated PASP [30]. However, this study included a relatively low number of non-*JAK2* mutated patients, and further studies are needed.

## 3. Clinical Evaluation and Management

### 3.1. Signs and Symptoms

Early recognition and surveillance of patients at risk or with symptoms of PH is imperative, though challenging given the often-nonspecific symptoms that can also be attributed to underlying malignancy. The most frequently observed symptoms include exertional dyspnea or exercise intolerance disproportionate to the degree of anemia or hematologic disease burden, unexplained hypoxia, syncope or presyncope, hemoptysis, weight gain due to fluid retention, or declining functional status [5]. Signs of PH that should be surveilled for on routine MPN follow-up include central or peripheral cyanosis, accentuated P2 heart sound, tricuspid regurgitation murmur, right heart strain or right bundle branch block on electrocardiogram, diastolic murmur of pulmonary insufficiency, ascites, hepatomegaly, peripheral edema, or abdominal distention [5].

Screening for PH in MPN patients should be performed using TTE in a risk-stratified rather than universal manner. Patients with newly diagnosed MPN, particularly those with MF, cardiopulmonary symptoms, splenomegaly, anemia, older age, or another feature associated with a high-risk of PH should undergo baseline TTE. Otherwise, those with established PV or ET should be screened based on the development of new symptoms or at disease milestones such as secondary progression [3,31]. Notably, the American Society of Echocardiography 2025 guidelines explicitly list MPNs as a condition warranting screening for PH, though those with MF may require a lower threshold and more regular screening interval given the higher prevalence of PH in this subgroup [3,32]. Similarly, the National Comprehensive Cancer Network guidelines recommend symptom assessment at baseline and during treatment for all MPN patients [1].

### 3.2. Management of Pulmonary Hypertension in Patients with Myeloproliferative Neoplasms

While data elaborating the epidemiology, pathophysiology, and clinical implications of PH in MPN patients are emerging, there is limited prospective data to guide management. Therefore, multidisciplinary collaboration is essential for comprehensive therapy for MPN patients with PH, as well as attaining greater clinical insight into the MPN/PH mechanism, trajectory, and future directions in management. As with all PH patients without MPN, the management of PH is tailored to the PH mechanism and addresses the underlying MPN with cytoreduction therapies, anticoagulation, allogeneic stem cell transplants, as indicated [23]. While PH-directed therapies like endothelin receptor antagonists, phosphodiesterase-5 inhibitors, and prostacyclin analogs are established therapies for Group 1 PAH, and soluble guanylyl cyclase stimulators for Group 4/CTEPH, robust data are not yet established in the treatment of pre-capillary Group 5 PH [33]. Patients with CTEPH may require additional interventions such as therapeutic anticoagulation, pulmonary artery endarterectomy, balloon pulmonary angioplasty, or riociguat in refractory cases, whereas general supportive care measures such as supplemental oxygen, diuretics, and avoidance of hypoxia or high-altitude are globally recommended [5].

The American College of Cardiology, American Heart Association, and American College of Chest Physicians recommend that all patients with PAH or CTEPH be evaluated at PH centers of excellence for collaborative care involving cardiologists, pulmonologists, hematologists, transplant physicians, and allied health professionals [34]. Early referral should be considered for patients with unexplained dyspnea, abnormal echocardiographic findings, or high-risk features (MF, splenomegaly), and should engage transplant teams early for MF patients with advanced disease. When PH is confirmed, a systematic workup should include assessment for autoimmune diseases, ventilation-perfusion scanning to exclude CTEPH, evaluation for LHD, and review of medication history for drug-induced causes [5,31]. There is scarce data for the impact of cytoreduction and cytoreductive therapies on MPN-associated PH. However, a retrospective study of patients with MF suggested that ruxolitinib, a *JAK1/2* inhibitor, may be associated with a lower risk of incident heart failure and PH, though larger, prospective studies are needed [35].

A potential treatment for MPN-associated PH is sotatercept. Sotatercept is associated with improved outcomes among patients with PAH and is a disease-modifying agent by inhibition of the activin–transforming growth factor (TGF)-SMAD pathway [24,25,26]. Additionally, sotatercept and a closely related drug, luspatercept, have been studied in patients with MF as a treatment for anemia via counteracting SMAD2/3-mediated suppression of terminal erythropoiesis [36,37]. The recent phase 2 CADENCE trial demonstrated hemodynamic benefit and early signs of clinical efficacy of sotatercept among patients with Cpc-PH, though phase 3 trials are needed to confirm clinical effectiveness of sotatercept on hard outcomes, including death, in Cpc-PH [38]. A case series of two patients with MPN-associated PH showed hemodynamic benefit after treatment with sotatercept [39]. However, prospective studies in this patient population are needed to confirm these findings and to evaluate the clinical efficacy of sotatercept in patients with MPN and PH.

## 4. Clinical Outcomes and Prognostic Value of PH in MPNs

Baseline patient characteristics, hemodynamic data, clinical outcomes, and the prognostic value of PH (primarily defined echocardiographically) in MPN patients are primarily derived from retrospective multicenter registry data [3]. Female sex is linked to lower rates of hematologic progression regardless of PH probability on echocardiography, though the impact on overall survival varies among registries, and PH status does not appear to be consistently associated with either sex or any race [4]. MPN patients with high echocardiographic probability of PH have higher baseline predisposition to other cardiovascular or multisystem comorbidities, including heart failure and associated hospitalizations, hypertension, atrial fibrillation, and chronic kidney disease [3]. Serologic data link the incidence of high echocardiographic probability of PH in MPN patients with higher median values of white blood cells, hemoglobin, creatinine, spleen size, and lactate dehydrogenase, suggesting an association with hematologic disease severity [3]. MF demonstrates the highest prevalence of high echocardiographic probability of PH (39.1% to 72.3%), with PV and ET exhibiting 10% prevalence [40]. The presence of high echocardiographic probability of PH in patients with MPNs has been associated with a longer duration of MPN diagnosis [3,11]. At the time of first TTE, patients with an intermediate-high echocardiographic probability of PH are more likely to have an advanced MPN phenotype, including higher rates of MF (24.5% vs. 10%), relative to those without PH. Similarly, baseline patient data from registries show that patients with high echocardiographic probability of PH had a longer time from MPN diagnosis to first TTE, with a median of nearly 50 months after MPN diagnosis versus nearly 33 months in those without PH [3,4].

In a large registry of patients with RHC-confirmed PH with MPN, the observed mortality was 30–40% specifically in patients with groups 4 and 5 PH, with the latter portending poorer 1-year survival (67% vs. 81%) [31]. In another retrospective registry of MPN patients who had undergone TTE, an association was demonstrated between high echocardiographic probability of PH and increased risk of hematologic progression to secondary MF or acute leukemia, as well as MACE (arterial or venous thrombosis, CHF hospitalization, or cardiovascular death) in MPN [3]. In a subgroup of patients who underwent RHC, there was an association between PH and increased risk of hematologic progression (29.4% versus 0%) [4]. Those with established cardiovascular disease, such as LHD, are further predisposed to a greater risk of all-cause death and cardiovascular death [41].

Prior studies suggest that LHD is common among patients with MPN [2]. Among patients with MPN, those with high echocardiographic probability of PH were more likely to have LHD, including increased rates of reduced left ventricular ejection fraction (<50%), left and right atrial dilation, left ventricular hypertrophy, and valvular disease [3]. Interestingly, the absence of LHD in patients with high echocardiographic probability of PH was associated with a higher risk of secondary MF progression among patients with ET or PV and of leukemia progression among patients with MF, suggesting differing pathophysiology in PH between patients with and without LHD in MPNs [3,4]. Collectively, these data suggest that when PH is attributable to LHD, the elevated pressures may partly reflect a common comorbidity rather than intrinsic MPN-driven pulmonary vascular disease. Conversely, PH without LHD likely reflects MPN-specific mechanisms such as EMH, inflammation, and vascular remodeling, which are more closely linked with hematologic progression.

In addition to the association with MACE and hematologic progression, PH is critical when considering patients for allogeneic hematopoietic cell transplant for MF. In one study of MF, 57% of patients with a high echocardiographic probability of PH had a higher rate of non-relapse mortality (21% vs. 7.1%), with most occurring within 100 days post-transplant, and lower overall survival (58.9% vs. 88.8%) [42]. Accordingly, the American Heart Association pre-transplant cardiovascular evaluation consists of initial risk stratification, exclusion of high-risk cardiovascular diseases, assessment of cardiac reserve, and reserve optimization through standard testing such as TTE and pulmonary function tests [43]. Median pulmonary artery systolic pressure decreases after transplant from a mean of 37 mmHg to 30 mmHg, and while statistically significant in the small series that reported this, it is unclear if this translates to a difference in outcomes [43]. Additional studies are needed to characterize the hemodynamic phenotypes of PH in MPN patients, as well as the hemodynamic and clinical impact of PH therapy.

## 5. Conclusions and Future Directions

There is a compelling bidirectional relationship between cardiovascular disease and MPN, as demonstrated through the association between PH, heart failure, and hematologic progression, and conversely MPN progression with worsening cardiovascular outcomes. PH in MPN is multifactorial and heterogenous; however, it is an important risk factor for MACE and hematologic progression across all MPN subtypes. Clinicians caring for patients with MPN should maintain a high index of suspicion for PH to ensure early screening and detection, appreciate the prognostic and therapeutic implications of PH to inform patient counseling and management, and have a low threshold for early multidisciplinary engagement.

Future prospective studies are necessary to identify optimal screening protocols and biomarkers for PH in MPN patients, inform risk stratification and prognostication models, identify patients appropriate for invasive hemodynamic evaluation, and determine to what extent PH-directed therapies may modify hematologic outcomes.

## Figures and Tables

**Table 1:** WHO classification of pulmonary hypertension.

PH Type	Etiologies	Prevalence	Therapeutic Strategy	Hemodynamic Profile
Group 1: Pulmonary arterial hypertension (PAH)	Idiopathic, heritable, drug- and toxin-induced, HIV, connective tissue disease, congenital heart disease, schistosomiasis, portal hypertension, persistent PH of newborn	Rare	Pulmonary vasodilators, calcium channel blockers in responders, lung transplant	Pre-Capillary
Group 2: Left heart disease-associated	HFpEF, HFrEF, valvular heart disease, congenital heart disease	Very common	Treatment of left heart disease	Isolated Post-Capillary
Group 3: Lung disease-associated	Obstructive and restrictive lung disease, hypoxia without lung disease, developmental lung disorders	Common	Treatment of underlying lung disease	Pre-Capillary
Group 4: Pulmonary artery obstruction-associated	Chronic thromboembolic disease or other pulmonary artery obstructions	Rare	PA endarterectomy, balloon angioplasty, riociguat	Pre-Capillary
Group 5: Multifactorial/unclear cause	Hematologic, systemic, and metabolic disorders	Rare	Treatment of underlying disease	Pre-capillary, isolated post-capillary, or Cpc-PH

Cpc-PH, combined pre- and post-capillary pulmonary hypertension; HFpEF, heart failure with preserved ejection fraction; HFrEF, heart failure with reduced ejection fraction; HIV, human immunodeficiency virus; PA, pulmonary artery; PH, pulmonary hypertension.

## Data Availability

No new data were created or analyzed in this study. Data sharing is not applicable.

## Abbreviations

The following abbreviations are used in this manuscript:

CHIP	Clonal hematopoiesis of indeterminate potential
CTEPH	Chronic thromboembolic pulmonary hypertension
ESC	European Society of Cardiology
ERS	European Respiratory Society
EMH	Extramedullary hematopoiesis
ET	Essential thrombocythemia
HF	Heart failure
HOHF	High-output heart failure
MACE	Major adverse cardiac events
MF	Myelofibrosis
mPAP	Mean pulmonary artery pressure
MPN	Myeloproliferative neoplasms
PCWP	Pulmonary capillary wedge pressure
PV	Polycythemia vera
PH	Pulmonary hypertension
RHC	Right heart catheterization
TTE	Transthoracic echocardiography
WHO	World Health Organization
WU	Wood units
